# Dynamics of Elastic Beams with Embedded Fluid-Filled Parallel-Channel Networks

**DOI:** 10.1089/soro.2014.0020

**Published:** 2015-03-01

**Authors:** Yoav Matia, Amir D. Gat

**Affiliations:** Faculty of Mechanical Engineering, Technion—Israel Institute of Technology, Technion City, Haifa, Israel.

## Abstract

A pressurized fluid-filled parallel-channel network embedded in an elastic beam, asymmetrically to the neutral plane, will create a deformation field within the beam. Deformation due to embedded fluidic networks is currently studied in the context of soft actuators and soft-robotic applications. Expanding on this concept, configurations can be designed so that the pressure in the channel network is created directly from external forces acting on the beam, and thus can be viewed as passive solid–fluid composite structures. We approximate the deformation of such structures and relate the fluid pressure and geometry of the network to a continuous deformation-field function. This enables the design of networks creating steady arbitrary deformation fields as well as to eliminate deformation created by external time-varying forces, thus increasing the effective rigidity of the beam. In addition, by including the effects of the deformation created by the channel network on the beam inertia, we can modify the response of the beam to external time-varying forces. We present a scheme to design channel networks that create predefined oscillating deformation patterns in response to external oscillating forces. The ability to include inertial effects is relevant to the design of dynamic soft robots and soft actuators. Our results are illustrated and validated by numerical computations.

## Introduction

Fluid enclosed within an elastic solid may apply pressure and shear stress on the fluid–solid interface and thus create a stress field and a deformation field within the solid.^1–4^ The interaction between the pressure field of an internal fluid-filled channel network and the deformation field of the supporting elastic structure is currently researched within the context of soft robotics and soft actuators.^5–15^ In this work we expand on the concept of pressurized soft actuators and suggest utilizing pressurized parallel-channel networks to significantly increase the effective rigidity of elastic structures by canceling deformation fields created by steady or time-varying external forces. The pressure within the channel network can be applied directly by the external forces (e.g., by a pin in contact with the fluid), and the structure thus can be viewed as a passive solid–fluid composite structure.

Currently, the majority of research on soft robots and soft actuators, based on fluidic network, focuses on experimental studies and quasi-static motions.^5–15^ The goal of this work is to provide a scheme to analyze and design embedded fluidic networks in order to create a predefined dynamic motion of a soft actuator. We will obtain a relation between the geometry of the channel network, the inertial effects, elastic stresses, and the time-varying deformation of the actuator. Such a relation will enable the design of rapidly moving soft robots and the inclusion of the effect of external loads on the performance of a soft actuator. While many works in the field use inhomogenous structures and large deformations, for simplicity we will focus here on a homogenous Euler–Bernoulli beam and assume small deformations as a model for the elastic and inertial dynamics of a soft actuator. These assumptions limit the validity of the model; however, for a given configuration with known properties, the results presented in this work can be readily extended.

**FIG. 1.**
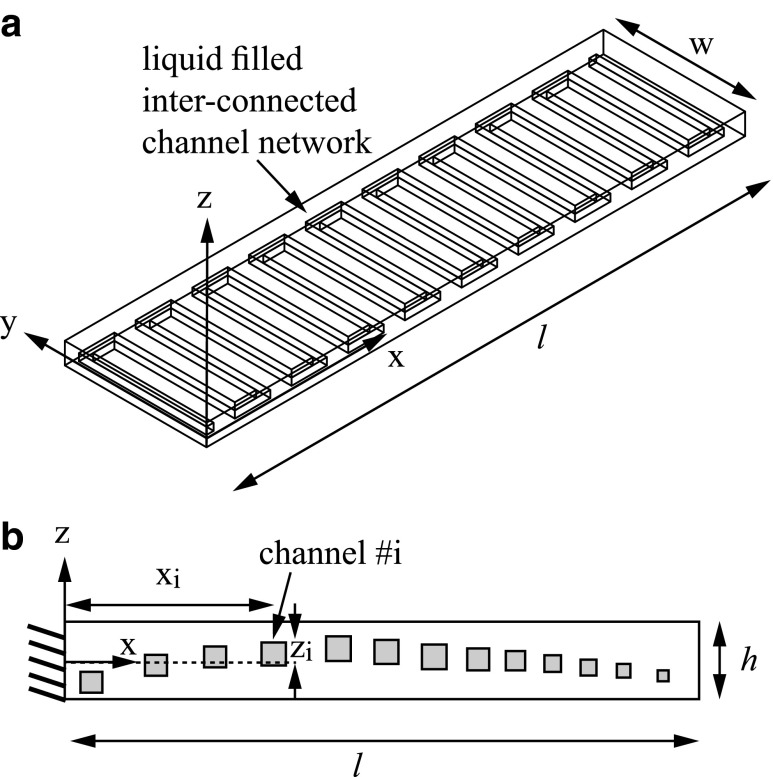
A three-dimensional illustration **(a)** and a cross-sectional illustration **(b)** of an elastic beam with an embedded interconnected parallel-channel network.

We study a rectangular beam with height *h*, width *w*, and length *l* under the requirements *h*/*w* « 1 and *w*/*l* « 1 (see Fig. 1a). The Young's modulus, density, Poisson's ratio, and mass per unit length of the beam are *E*, ρ, ν, and *μ_s_*, respectively. An interconnected parallel-channel network is distributed within the beam perpendicular to the *x* – *z* plane (see Fig. 1b). The fluid pressure is *p* and is assumed spatially uniform. The difference between the length of a single channel and the width of the beam *w* is required to be negligible compared with it's length *l*. The total length of channel segments connecting the parallel channels is required to be negligible compared with the total length of channel network. In addition, we focus on channel networks with negligible effect on the second moment of inertia and mass per unit length of the beam. The deflection of the beam in the *z* direction is denoted by *d*. We assume small deformations so that *d*=*d_e_*+*d_n_* is a sum of *d_e_*, the deformation due to external forces, and *d_n_*, the deformation due to the pressurized channel network.

## Analysis

**FIG. 2.**
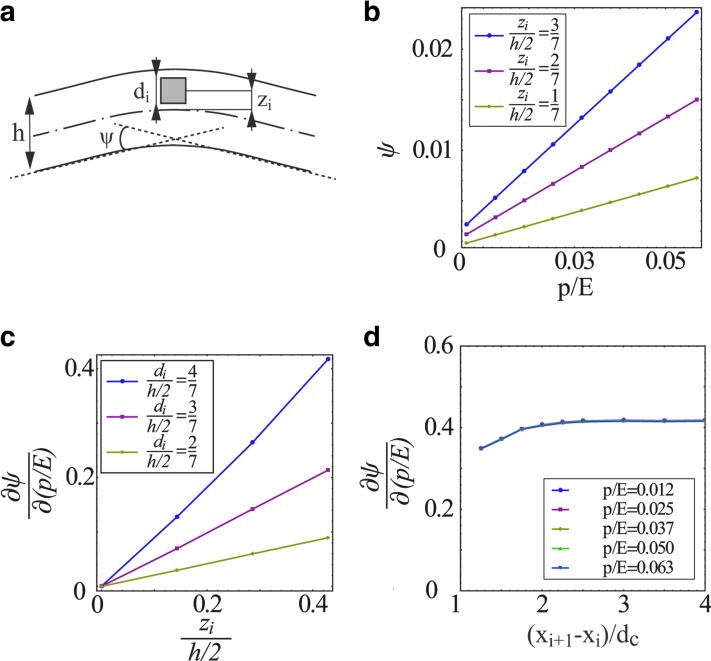
**(a)** The definition of *ψ* and the geometric parameters of the channel; **(b)** *ψ* vs. *p/E* for various values of *z_i_*/(*h*/2), where *d_i_/*(*h*/2)=4/7; **(c)** *∂ψ/∂*(*p/E*) vs. *z_i_*/(*h*/2)for various *d_i_*/(*h*/2); and **(d)** *∂ψ/∂*(*p/E*) vs. (*x_i_*_+1_–*x_i_*)/*d_i_*, the distance between centers of adjacent channels, for various *p/E*. In **(b)** and **(c)** the channel cross section is a square with width and height *d_i_*.

A single pressurized channel will create local stress and strain fields that will decay far from the channel.^16^ A pressurized channel positioned asymmetrically with regard to the midplane will create a change of the slope of the beam due to asymmetric strain field (see Fig. 2a). For a sufficiently small ratio *h*/*w* « 1, the problem is approximately two-dimensional, and thus we can define the change in beam slope due to a single channel as *ψ*:

\begin{align*} \frac { \partial d_n ( x_i + \Delta x ) }  {
\partial x } - \frac { \partial d_n ( x_i - \Delta x ) }  {
\partial x } = \psi \left( \frac { p }  { E } , v , \frac { z_i }
{ h } , \frac { d_i }  { h } \right) , \tag { 1 } \end{align*}

where *x_i_* is the location of the center of the channel and Δ*x* is sufficiently large so that the stress field vanishes. The value of *ψ*, the change in beam slope due to a single channel, can be obtained numerically or experimentally for a given material, pressure, and channel configuration.

Throughout this work we present numerical computations in order to validate our analysis. In all cases we simulate a beam with *h*=7·10^−3^ m, *w*=5·10^−2^ m, *l*=0.1 m, *E*=8·10^6^ Pa, *ρ*=3500 kg/m^3^, and ν=0.4. The channel cross section is square with width *d_i_* /(*h*/2)=4/7. The beam includes a 0.5 mm area on all sides without a network, and the connecting channels have identical properties to the parallel channels. A spatially uniform pressure is applied at the solid–fluid interface. Our computations utilize commercial code COMSOL™ multiphysics 4.3 with ≈10^5^ grid elements to calculate the solid deformation, using the Rayleigh damping algorithm with mass damping coefficient *α*=6.352·10^−3^ [1/s] and material structural damping coefficient *β*=1.136·10^−3^ [s].

In Figure 2 we present values of *ψ* obtained by numerical computations for a channel with a square cross section. Figure 2a illustrates the definition of *ψ* and the geometric parameters of the channel, including *z_i_*, the distance of the channel center from the midplane, and *d_i_*, the width and height of the square cross section. Figure 2b presents *ψ* versus *p*/*E* for various values of *z_i_*/(*h*/2), where *d_i_*/(*h*/2)=4/7. Figure 2c presents *∂ψ*/*∂*(*p*/*E*) versus *z_i_*/(*h*/2) for various *d_i_*/(*h*/2). Figure 2b and c show that *ψ* increases monotonically with *z_i_*/(*h*/2) and *d_i_*/(*h*/2). Figure 2d presents *∂ψ*/*∂*(*p*/*E*) versus (*x_i_*_+1_ – *x_i_*)/*d_i_* for various *p*/*E*, examining the effect of interaction between adjacent channels on *∂ψ*/*∂*(*p/E*). The influence of adjacent channels is shown to be small, even for distances of (*x_i_*_+1_ – *x_i_*)/*d_c_*≈1.2. From Figure 2a–c, the value of *ψ* is approximately linear with *p*/*E*, and thus

\begin{align*}\psi \approx \frac { p }  { E } \frac { \partial \psi }  { \partial ( p / E ) } \left( \frac { p }  { E } = 0 , \nu , \frac { z_i }  { h } , \frac { d_i }  { h } \right) . \tag { 2 } \end{align*}

We define the channel density φ of a parallel-channel network (see Fig. 1) as the number of channels per unit length. For characteristic length scale *l* much greater than the characteristic distance between the channels (*l* » 1/φ), we can approximate the change in slope to a continuous function:

\begin{align*} \frac { \partial^2 d_n }  { \partial x^2 } = \frac { 1 }  { dx } \left( \frac { \partial d_n ( x + dx ) }  { \partial x } - \frac { \partial d_n ( x ) }  { \partial x } \right) = \frac { 1 }  { dx } ( k \psi ) , \tag { 3 } \end{align*}

where *k* is the number of channels in the interval *dx*. Defining the local density of the channels as *φ*=*k*/*dx* and applying (2) yields a relation between the parallel-channel configuration and the deformation pattern created by the pressurized network, denoted as *d_n_*:

\begin{align*} \frac { \partial^2 d_n }  { \partial x^2 } = - \phi \frac { p }  { E } \frac { \partial \psi }  { \partial ( p / E ) } \left( \frac { p }  { E } = 0 , \nu , \frac { z_i }  { h } , \frac { d_i }  { h } \right) . \tag { 4 } \end{align*}

From Eq. (4) we can obtain the required geometry of a channel network to create a predetermined deformation field *d_n_*. After calculating *φ* from Eq. (4), the location of the center of the channel *x_i_* is determined by

\begin{align*}\int_ { 0 } ^ { x_ { i } } \mid \phi \mid dx = i - \frac { 1 }  { 2 } , \tag { 5 } \end{align*}

where *i* is a natural number. Hereafter, in all cases, we solve *φ* for *z_i_*/(*h*/2)=4/7. For cases in which we obtain *φ*<0 (negative channel density), we replace *z_i_*/(*h*/2)=4/7 with *z_i_*/(*h*/2)=−4/7 and thus change the sign of *∂ψ/∂ (p/E)*.

## Results

**FIG. 3.**
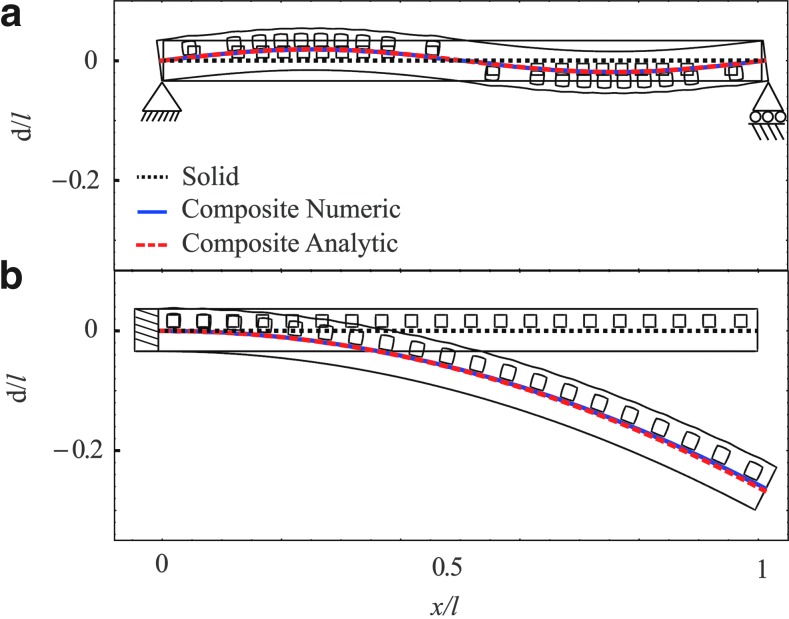
The deformation field created by a channel network calculated by Eq. (4) for *d_n_*/*l*=0.02 sin (2 π*x*/*l*) **(a)** and ${d_n / l} { = \sqrt{4 - ( {x / l} ) ^2} - 2}$ **(b)**. Composite solid–fluid deflection is marked by red dashed lines (analytic) and smooth blue lines (numeric). For comparison, deformation field without the network is marked by black dotted lines.

Figure 3 illustrates the creation of an arbitrary steady deformation field of the beam by designing the channel network according to Eq. (4). Figure 3a presents sine deformation field *d_n_*/*l*=0.02 sin (2*πx*/*l*) and Figure 3b presents a circular deformation defined by (*x*/*l*)^2^+(*d_n_*/*l*+2)^2^=4. Good agreement is observed between the model (red dashed lines) and numerical computations (blue solid lines).

For slender linearly elastic beam, the deformation created by steady external forces, denoted as *d_e_*, is given by Euler–Bernoulli beam theory as *∂*^2^ *d_e_*/*∂x*^2^=*M*/*EI*, where *M* is the bending moment and *I*=*h*^3^ *w*/12 is the second moment of inertia. Assuming small deformations, the total deflection of the beam is *d*=*d_n_*+*d_e_*. Thus, the deflection due to external forces, *d_e_*, can eliminated by requiring

\begin{align*} \frac { \partial^2 d_n }  { \partial x^2 } + \frac { \partial^2 d_e }  { \partial x^2 } = 0 \rightarrow - \frac { p ( t ) }  { E } \phi ( x ) \frac { \partial \psi ( x ) }  { \partial ( p / E ) } + \frac { M }  { EI } = 0. \tag { 6 } \end{align*}

Therefore, for any bending moment distribution that can be presented as *M*=*f*_1_(*t*) *f*_2_(*x*), the deflection field can be eliminated by requiring *p*(*t*)=*f*_1_(*t*) and *φ*(*x*)*∂ψ*(*x*)/*∂*(*p*/*E*)=*f*_2_(*x*). Since the total deformation *d*=*d_e_*+*d_n_* is constant, no inertial effects will be created due to the time-varying external forces.

**FIG. 4.**
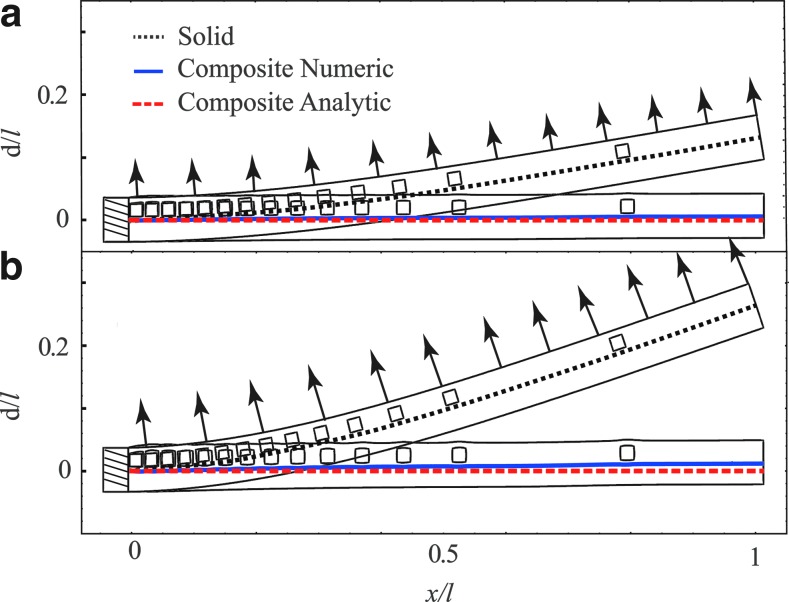
The deformation field created by a channel network calculated by Eq. (6) in order to cancel external uniform load. Two values of *q*/*E*=2.5·10^−5^ **(a)** and *q*/*E*=5·10^−5^ **(b)** are examined. Composite solid–fluid deflection is marked by red dashed lines (analytic) and smooth blue lines (numeric). For comparison, deformation field without the network is marked by black dotted lines.

In Figure 4 we illustrate utilizing an internal fluidic network to enhance the effective rigidity of an elastic beam. The required deformation field is marked by red dashed lines and the deformation obtained by numerical computations is marked by solid blue lines. For comparison, a solid beam without embedded channel network is presented by dotted black lines. For the case of uniform load *q*/*E*=2.5·10^−5^ (e.g., load acting on a wing), the required network geometry was calculated according to Eq. (6) for *p*/*E*=3.16·10^−2^. Since the deformation is linear both with *p/E* and with *q*/*E*, an increase of the external load to *q/E*=5·10^−5^ would be eliminated by a proportional increase in the network pressure to *p/E*=6.32·10^−2^. Thus, the cancellation of deformation by varying external load can be eliminated by a single network configuration.

**FIG. 5.**
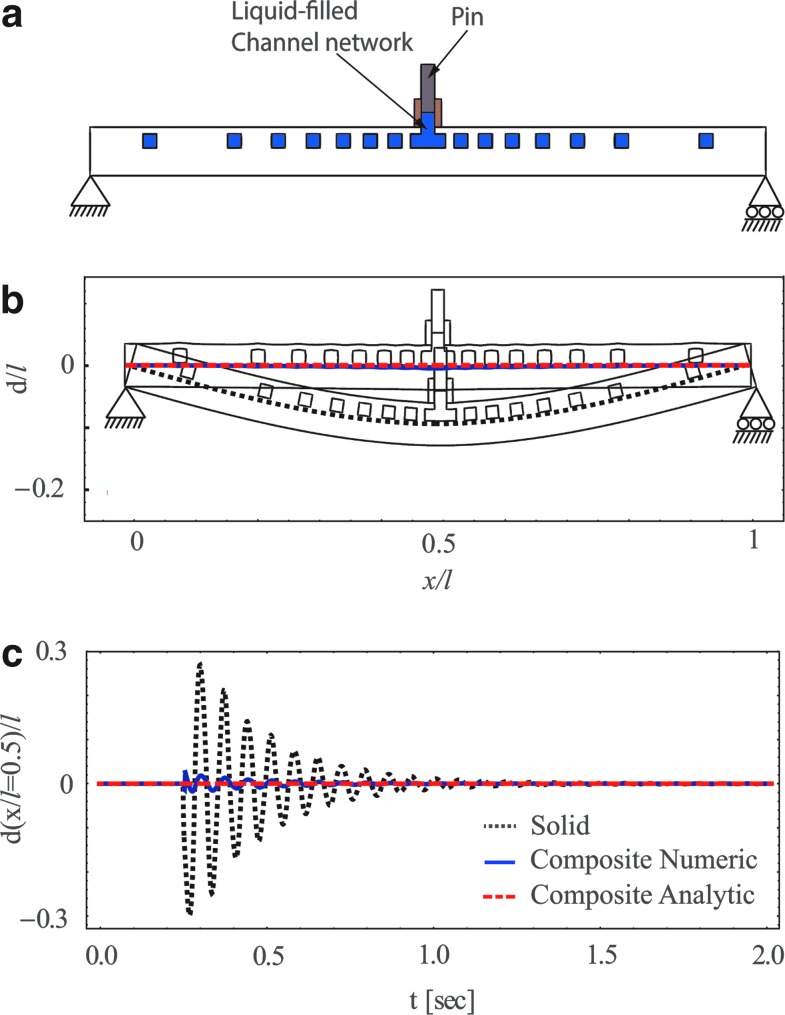
**(a)** A cross-sectional illustration of a solid–fluid composite structure. **(b)** The deformation field created by the external force *f* on the pin (where *f*=3.15 [N] and a=6.23·10^−6^ [m^2^]). **(c)** Response to an impulse *f*=δ(*t − t_s_*) 1.26·10^−2^ [N] where *t_s_*=0.35 [s]. The channel network is calculated by Eq. (4) in order to eliminate deformation. Composite solid–fluid deflection is marked by red dashed lines (analytic) and smooth blue lines (numeric). For comparison, deformation field without the network is marked by black dotted lines.

On the basis of the above, we suggest a fluid–solid composite structure in which application of external force directly creates pressure within an internal channel network. Such structures will allow control of the dynamic response of beams to external loads by the addition of the deformation created by the pressurized network configuration to the deformation created by external forces. An illustration of such a structure is presented in Figure 5a, where a force *f* may be applied by a pin directly on the fluid, creating a fluid pressure *p*=*f*/*a*, where α is the area of the pin. Figure 5 presents the response of such a structure to steady external force *f*=3.15[N] (Fig. 5b) and to a sudden impulse *f*=δ(*t*–*t_s_*) 1.26·10^−2^[N], where δ is Dirac's delta function (Fig. 5b). Order of magnitude reduction in deformation is observed for both the steady and time-varying external forces.

So far we focused on creating steady deformation fields. In order to create a predefined time-varying deformation field, the design of the internal channel network will need to include the effect of solid inertia. The deformation field created by the channel network yields acceleration of the beam, and thus the Euler–Bernoulli equation is

\begin{align*} \frac { \partial^2 }  { \partial x^2 } \left( EI \frac { \partial^2 d_e }  { \partial x^2 } \right) = - \mu_s \frac { \partial^2 }  { \partial t^2 } ( d_e + d_n ) + qw , \tag { 7 } \end{align*}

where *μ_s_* is beam mass per unit length. Substituting *d*=*d_e_*+*d_n_* and Eq. (4) yields equation of the total deflection including the effects of the channel network geometry and time-varying (spatially uniform) pressure as

\begin{align*} \frac { \partial^2 }  { \partial x^2 } \left[ EI \left( \frac { \partial^2 d }  { \partial x^2 } + \phi \frac { p }  { E } \frac { \partial \psi }  { \partial ( p / E ) } \right) \right] = - \mu_s \frac { \partial^2 d }  { \partial t^2 } + qw. \tag { 8 } \end{align*}

Solution of Eq. (8) can be obtained for an oscillating deformation of the form *d/l*=*D* (*x*) sin (*ωt*+*θ*) under similarly oscillating external load *q*=*Q* (*x*) sin (*ωt*+*θ*), where *D*(*x*) and *Q*(*x*) are known functions defining deformation and external load, respectively, *ω* is the angular frequency, and *θ* is the phase. For the case of a solid–fluid composite (see Fig. 5a), the internal pressure is proportional to the external force, and thus *p*=*P* sin (*ωt*), where *P* is a known constant. Substituting *d*/*l*, *q*, and *p* into Eq. (8) yields the required network density:

\begin{align*}\phi & = \left[ \int_0^x \int_0^ \eta ( \mu_s \omega_n^2 lD ( \xi ) - w Q ( \xi ) ) d \xi d \eta - l\frac{\partial^2 D}{\partial x^2} \right] \\ & \quad \times\left( P\frac{\partial \psi}{\partial (p / E)}EI \right)^{- 1}  \tag{9}\end{align*}

**FIG. 6.**
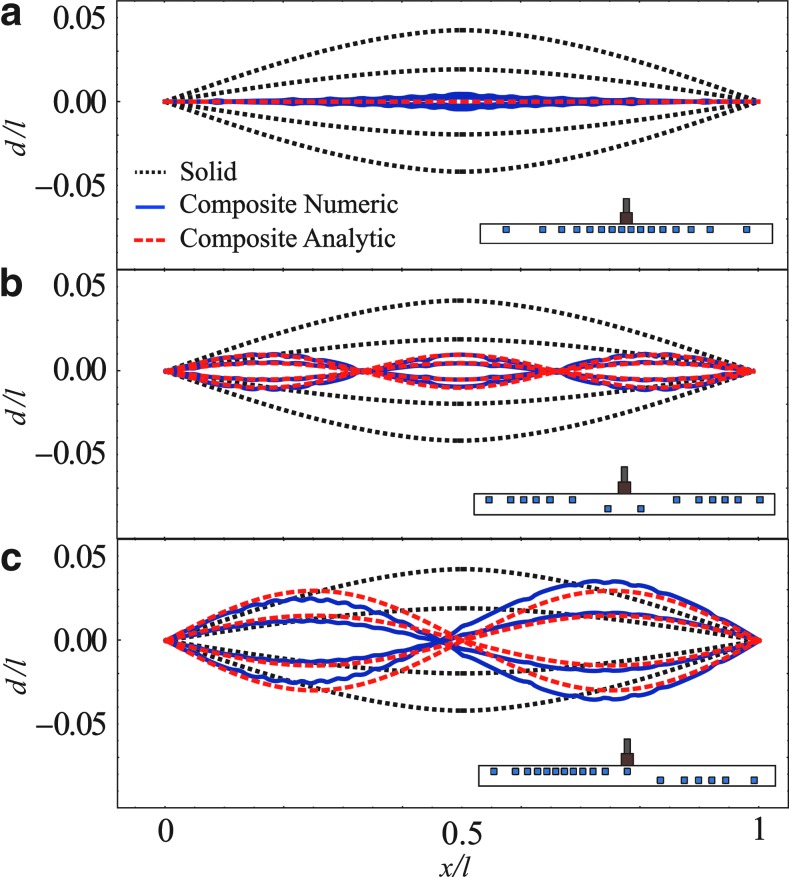
Deflection of a solid–fluid composite beam due to external oscillating force acting at *x*/*l*=0.5. The parallel-channel network (illustrated in inserts) is designed by Eq. (9) to create deflection **(a)** *d/l*=0, **(b)** *d*/*l*=0.01 sin (3π*x/l*) sin (*ωt*) and **(c)** *d/l*=0.03 sin (2π*x/l*) sin (*ωt*), where ω=62.8 [1/s]. Each time cycle is divided into four equal parts. Composite solid–fluid deflection is marked by red dashed lines (analytic) and smooth blue lines (numeric). For comparison, deformation field without the network is marked by black dotted lines.

We illustrate use of Eq. (9) for the case presented in Figure 5a with *q*/*E*=*C*_1_ δ (*x*/*l* – 1/2) sin (*ωt*)/*w*, and thus *p*/*E*=*C*_1_ sin (*ωt*) *wl/a*, where *a*=2.207·10^−6^ [m^2^] is the area of the pin and *C*_1_=2[N]. The value of *ω* is 62.8[1/s], where the natural angular frequency of the beam is ≈88[1/s]. Figure 6a presents the effects of oscillating external force for φ designed by Eq. (9). In part (a) *d*/*l*=0, in part (b) *d/l*=0.01 sin (3 π*x*/*l*) sin (*ωt*), and in part (c) *d/l*=0.03 sin (2 π*x*/*l*) sin (*ωt*). The required deformation field is marked by red dashed lines and the deformation obtained by numerical computations is marked by solid blue lines. Good agreement is observed between the theoretic predictions and the numerical computations.

## Concluding Remarks

In order to apply this work to specific experimental configuration, the actuator geometry needs to be approximated to an Euler–Bernoulli beam and the change of angle due to a single channel as a function of pressure, *ψ*(*p*), needs to be obtained from experimental or numerical data. For small deformations, the required parameters are Young's modulus *E* second moment of inertia *I*, mass per unit length *μ_s_*, and the value of *∂ψ*/*∂p* at *p*=0. While in this work, for simplicity, we presented solutions for cases of homogenous beams with a constant cross section, Eqs. (1–8) are also valid for inhomogeneous beams with spatially varying *∂ψ*/*∂p* (*x*), *E*(*x*), *I*(*x*), and *μ_s_*(*x*). For configurations where the approximation *ψ(p)≈p∂ψ*/*∂p* is incorrect, Eq. (8) still describes the dynamics of the beam, as long as the Euler–Bernoulli approximation is valid. However, the term *p∂ψ*/*∂p* should be replaced with *ψ*(*p*) and the effect of the pressure on the moment of inertia *I*(*p*) and mass per unit length *μ_s_*(*p*) should be included. For such cases the governing equation is expected to be nonlinear and would thus require a specific mathematical treatment based on the type of nonlinearity.

In conclusion, embedded fluidic networks can be used to create complex time-varying deformation patterns in elastic beams. We presented a scheme to control such time-varying and periodic deformation fields of soft actuators via the geometry of the embedded fluidic network. The ability to design time-varying deformation field may be used as the basis for the design of running, jumping, or any maneuvering soft robots with nonnegligible inertial effects. We also present a scheme to utilize external forces acting on the actuator to directly pressurize the channel network (as presented in Fig. 5a). Such configurations allow for passive control of the response of the beam to external loads, and can be viewed as composite solid–fluid structures. Future research may include the effects of fluid viscosity and a nonuniform pressure distribution on the transient response of such structures to external forces, as well as modeling the complete dynamics of a maneuvering robot actuated by a combination of elastic beams with fluidic embedded channel networks.
